# *Spiroplasma*, *Wolbachia*, *Sodalis* and trypanosome associations in *Glossina Tachinoides* from Yankari game reserve, Nigeria

**DOI:** 10.1186/s12917-025-04959-7

**Published:** 2025-08-13

**Authors:** Atoh Cedric Munu Tamuton, Youssouf Mouliom Mfopit, Aminu Bashir Yusuf, Peter Yunenui Mahbou, Edwige Flore Gouegni, Grace Amarachi Amos, Mohammed Mamman, Auwal Adamu, Gloria Dada Chechet, Junaidu Kabir

**Affiliations:** 1 Africa Centre of Excellence for Neglected Tropical Diseases and Forensic Biotechnology, Ahmadu Bello University, Zaria, Nigeria; 2 Department of Biochemistry, Ahmadu Bello University, Zaria, Nigeria; 3 Institute of Agricultural Research for Development (IRAD), Yaounde, Cameroon; 4 Nigerian Institute for Trypanosomiasis Research (NITR), Kaduna, Nigeria; 5 Department of Zoology, Ahmadu Bello University, Zaria, Nigeria; 6 Department of Veterinary Pharmacology and toxicology, Ahmadu Bello University, Zaria, Nigeria; 7 Department of Veterinary Public Health and Preventive Medicine, Ahmadu Bello University, Zaria, Nigeria; 8 Africa Centre of Excellence for Mycotoxins and Food Safety, Federal University of Technology, Minna, Nigeria; 9 College of Pharmacy and Pharmaceutical Sciences, Institute of Public Health, Florida Agricultural and Mechanical University, Florida, United States of America

**Keywords:** *Trypanosoma*, *Spiroplasma*, *Wolbachia*, *Sodalis glossinidius*, *Glossina Tachinoides*, Yankari game reserve

## Abstract

**Background:**

Tsetse flies are vectors of African trypanosomiasis, a disease that affects both humans and animals. Trypanosomiasis remains a threat to lives and it is an impediment to socio-economic development in sub-Saharan Africa. In spite of decades of chemotherapy and vector control, the disease has not been eradicated. Parasitic drug resistance has been developed to existing drugs, while vector control strategies are expensive and unsustainable. Therefore, there is a need to explore other control approaches, such as the transformation of tsetse fly endosymbionts to render the fly refractory to trypanosome infection. This research focused on investigating the prevalence and triparty association of infection of trypanosomes with some endosymbionts of tsetse flies from Yankari Game Reserve.

**Methods:**

Tsetse flies were captured using biconical traps, identified morphologically, dissected and their entire guts were isolated and used for DNA extraction. Polymerase Chain Reaction (PCR) was used in confirming the identity of the tsetse flies by amplifying the cytochrome C oxidase-1 gene. PCR was also used to screen for the presence of endosymbionts (*Sodalis glossinidius*, *Wolbachia*, and *Spiroplasma* sp.) and trypanosomes.

**Results:**

*Glossina tachinoides* was the only vector species identified. Trypanosome infection rate was 10.70% with *Trypanosoma grayi* being the most prevalent (9.78%) amongst the three trypanosome species detected. The prevalence of *Wolbachia* and *Spiroplasma* species were 2.80% and 40.8% respectively in flies. *Sodalis glossinidius* was not detected. There was an association between the presence of trypanosomes and *Wolbachia*, while no association was depicted between trypanosomes and *Spiroplasma*.

**Conclusion:**

It has been observed from this study that the presence of *Wolbachia* seems to favour trypanosome infections. Investigation on the *Wolbachia* genetic polymorphism in tsetse could help to better understand this association.

**Supplementary Information:**

The online version contains supplementary material available at 10.1186/s12917-025-04959-7.

## Introduction

Tsetse flies are viviparous obligate hematophagous insects found in sub-Saharan Africa serving as vectors of African trypanosomiasis, a parasitic disease known as Human African Trypanosomiasis (HAT) in humans and Animal African Trypanosomiasis (AAT) in animals [1]. AAT affects wild fauna, domestic animals (goats, dogs, sheep and pigs) and domesticated livestock like camels, donkeys and horses [2]. Economic retardation due AAT is estimated at 4.75 billion dollars annually [3]. Vector control and drug administration are being used to control AAT. Drugs presently used in the management of trypanosome infections in animals are less efficient due to the increasing number of drug resistant strains, high cost, toxicity, and less availability [4]. Trapping, use of insecticides and sterile insect control is used for vector control. However, the drawbacks of these techniques are their field application and sustainability which include limited resources for trapping and development of resistance to insecticides. In addition, most insecticides are not environmentally friendly. Sterile insect technique is specific to a particular species of tsetse flies [5].

Investigations of endosymbionts that could be implicated in the vector competence of tsetse flies have been undertaken in different tsetse species [6]. *Sodalis glossinidius* have been genetically transformed to express anti-trypanosome molecules within tsetse flies making them refractory to trypanosomes, a technique known as paratransgenesis [7]. Mitigating vector competence will require complete interruption of disease transmission. These could be achieved using genetic engineering to generate transgenic insects capable of blocking the biological and cyclical transmission of the parasites [8]. The microbial community influences several aspects of Tsetse’s physiology, including nutrition, fecundity, and vector competence [9, 10]. Selected strains of this microbial community prevent the vector from transmitting diseases by either blocking pathogen development or reducing the life span of the vector [11]. Tsetse flies harbour three maternally transmitted symbiotic bacteria: *Wolbachia*, *Sodalis glossinidius* and *Wigglesworthia glossinidia* [10, 12]. Recently, *Spiroplasma* was reported as an endosymbiont of tsetse flies [13].

*W. glossinidia* is an obligate symbiotic bacterium found in all tsetse species. It provides food supplements to maintain fecundity. It is also important in the maturation of the immune system of the fly [6], however, not relevant for this current study. *S. glossinidius* is involved in tsetse fly vector competence by favouring parasite fixing in the insect midgut via a biochemical mechanism involving the production of N-acetyl glucosamine [12]. The colonisation of the tsetse’s midgut and spread of the trypanosome correlate positively with the presence of *S. glossinidius* [14]. *Wolbachia* is an intracellular alpha proteobacteria that is trans-ovarially transmitted and occur in about 65% of arthropods, associated with reproductive tissues and cause reproductive abnormalities such as cytoplasmic incompatibility (CI) and parthenogenesis [15]. *Spiroplasma* is a genus of wall-less bacteria belonging to the class *Mollicutes* and has been shown to protect *Drosophila neotestacea* from nematode infection [16] against fungi in the pea aphid (*Acyrthosiphon pisum*), and against a parasitoid wasp in *Drosophila hydei* [17]. Reports are available on the prevalence of *S. glossinidius*, *Wolbachia*, and *Spiroplasma* in tsetse species from different communities across the globe highlighting an association between the presence of the endosymbiont *S. glossinidius* and the aptness of tsetse flies to harbour trypanosomes [18]. Despite these reports, data is lacking on the association between endosymbionts and trypanosomes in *G. tachinoides* from Yankari Game Reserve, Nigeria, a region with tourism and agriculture as some major income-generating activities. Therefore, this study aimed to investigate the prevalence and association between trypanosomes and endosymbionts in tsetse flies from Yankari Game Reserve.

## Methods

### Study area

**Fig. 1:**
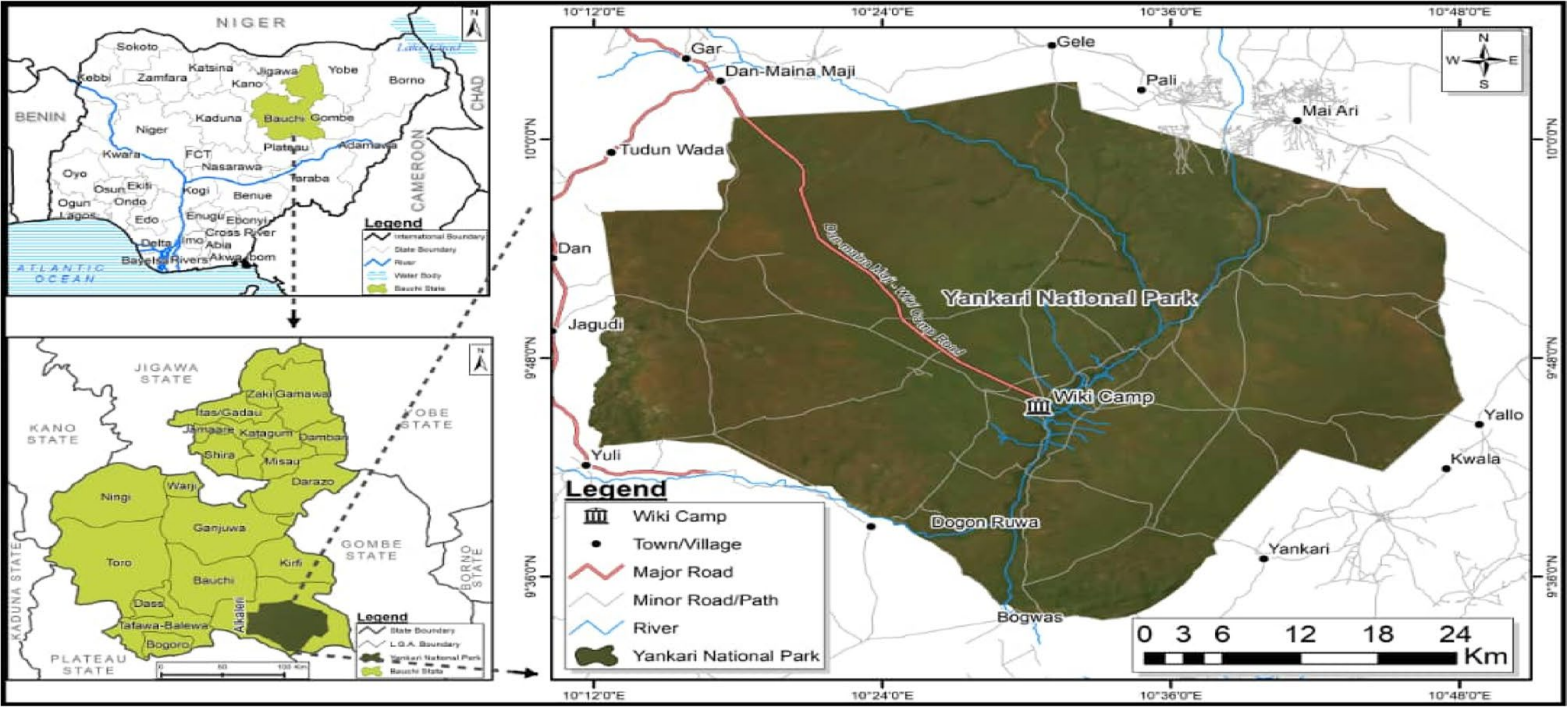
Study area

This study was done in Yankari Game Reserve (Fig. 1). Yankari Game Reserve is known to have a lot of tsetse flies and also harbour wildlife that could act as reservoirs of African Trypanosomiasis. In addition, Yankari Game Reserve is a region with tourism and agriculture as some major income-generating activities. Thus, controlling trypanosomiasis is this region will minimise economic losses. The game reserve occupies an area of about 2244 km^2^. The park is centred at 9.50’N and 10.30’E in the South-central area of Bauchi State in North-eastern Nigeria [19]. The annual rainfall in the area ranges between 900 mm^3^ and 1,000 mm^3^, with the wet season starting in May and ending in September and the dry season starting in October and ending in April [20]. Its moderate climate, dense vegetation providing shelter, presence of slow-flowing streams, and wild animals make the environment suitable for tsetse flies. The rich diversity of animals in the reserve probably constitutes the main food source for the tsetse population.

### Sampling and morphological identification

Trapping of tsetse flies was carried out in November using 11 biconical traps placed at suitable locations (cool, shady areas to avoid desiccation of flies) for three days consecutively. The coordinates of all traps were recorded using a Global Positioning System (GPS) device (GPSMAP^®^ 60CSx Garmin). Collection of tsetse flies was once daily at 4 pm. The species of tsetse flies was identified immediately using morphological features such as size, colour and number of dark tarsal segments following the Food and Agricultural Organization (FAO) training manual for tsetse personnel volume 1 [21] while sex was determined based on the presence or absence of hypogeum [22]. The flies were dissected under a microscope in a drop of phosphate buffered saline (PBS) solution using sterile dissection tools. The tools were cleaned using 3% bleach (sodium hypochlorite), followed by 70% ethanol, and finally sterile distilled water after the dissection of each fly to prevent contamination. Guts and other body parts were separately transferred into cryotubes containing RNA Later^®^ (Sigma-Aldrich) and kept at room temperature in the field, then at − 20 ℃ in the laboratory until required for use.

### DNA extraction

Nucleic acid material (DNA) was isolated from the guts using AccuPrep Genomic DNA extraction kit (Bioneer, South Korea) according to the instructions of the manufacturer. The extracted DNA was quantified using a NanoDrop1000 C spectrophotometer (Thermo scientific, Germany) and stored at −20 ℃.

### Molecular confirmation of tsetse fly species

To confirm the species of tsetse flies previously identified morphologically, a PCR was performed to amplify the mitochondrial *cytochrome C oxidase 1 gene* (COX1) using COX-1 primers (Table 1) adapted from Dyer et al. [23]. The PCR reaction was performed in a 20 µL volume containing 1X DreamTaq buffer, 1 U DreamTaq polymerase, 0.2 mM dNTPs, 2 µM of forward and 2 µM of reverse primers, 2 µL of DNA template, and the final volume was made up with nuclease-free water. The cycling conditions were; initial denaturation at 95 ℃ for 5 min, followed by 30 cycles of denaturation at 94 ℃ for 60 s, annealing at 55 ℃ for 60 s, and elongation at 72 ℃ for 2 min. The final extension was at 72 °C for 10 min.

### Identification of trypanosome species

A nested PCR using ITS-1 generic primers (Table 1) was performed according to Adams et al. [24]. The first round of PCR was performed in a 20 µL reaction volume containing 1X DreamTaq buffer, 1 U DreamTaq polymerase 0.2 mM dNTPs, 1 µM each of the outer primers (forward and reverse), and 3 µL of DNA template. The volume was made up using nuclease free-water. The cycling conditions were; initial denaturation at 95 ℃ for 3 min, followed by 35 cycles of denaturation at 94 ℃ for 30 s, annealing at 54 ℃ for 30 s, extension at 72 ℃ for 60 s, with a final extension at 72 ℃ for 5 min. The second round of PCR was carried out using ITS-1 internal primers (Table 1) and was performed in 20 µL reaction volume containing 1X DreamTaq buffer, 1 U DreamTaq polymerase, 0.2 mM dNTPs, 1 µM of each primer, 2 µL DNA (1/40 dilution of first round PCR product) and the volume was made up with nuclease free water. The cycling conditions were as follows: initial denaturation at 95 ℃ for 3 min, followed by 35 cycles of denaturation at 94 ℃ for 30 s, annealing at 55 ℃ for 30 s, extension at 72 ℃ for 60 s, and a final extension at 72 ℃ for 5 min. The expected band sizes for trypanosome species were: *Trypanosoma congolense* (640 bp), *Trypanosoma grayi* (240–380 bp), *Trypanosoma vivax* (400 bp), *Trypanosoma simiae* (400 bp), *Trypanosoma brucei* (500 bp) and *Trypanosoma evansi* (500 bp).

Another nested PCR targeting an 800 bp DNA fragment of the glycosomal Glyceraldehyde-3-phosphate dehydrogenase (gGAPDH) gene of trypanosomes was performed to confirm the trypanosomes positive tsetse guts using gGAPDH primers (Table 1) designed by Hamilton et al. [25] following the protocol of Weber et al. [26]. The first round PCR was performed in a 20 µL reaction volume containing 1X DreamTaq buffer, 1 U DreamTaq polymerase, 0.2 mM dNTPs, 1 µM of each gGAPDH external primer, 2 µL DNA template and the volume was made up using nuclease free water. The cycling conditions were; initial denaturation for 3 min at 95 °C, followed by 30 cycles of 60 s at 95 °C, 30 s at 55 °C and 60 s at 72 °C, and a final elongation at 72 °C for 10 min. The first PCR products were diluted 40-fold and 1 µL of this dilution was used for the second PCR round using gGAPDH internal primers, performed in a 20 µL reaction volume containing 1X DreamTaq buffer, 1 U DreamTaq polymerase, 0.2 mM dNTPs, 1 µM each of gGAPDH internal primer (Table 1), and the volume was made up using nuclease free water. The cycling conditions were; initial denaturation for 3 min at 95 °C, followed by 30 cycles of 60 s at 95 °C, 30 s at 52 °C and 60 s at 72 °C, and a final elongation at 72 °C for 10 min. Amplicons were resolved on 1.5% agarose gel. The double distilled water was used as negative control while trypanosome positive controls were obtained from Weber et al. [26] DNA positive samples.

### Detection of endosymbionts

The presence of *S. glossinidius* was screened by PCR, amplifying a 120 bp DNA fragment of *S. glossinidius* extrachromosomal plasmid (pSG2) using pSG2 primers (Table 1) adapted from Darby et al. [27] in a total reaction volume of 20 µL containing 1X DreamTaq buffer, 1 U DreamTaq polymerase, 0.2 mM dNTPs, 1 µM each of primers, and 3 µL of DNA template. The volume was made up using nuclease-free water. The cycling conditions were: initial denaturation at 94 ℃ for 3 min, followed by 30 cycles of 94 ℃ for 30 s, 51 ℃ for 30 s, 72 ℃ for 30 s, and a final extension at 72 ℃ for 5 min. *Wolbachia* was detected by PCR using W-spec primers (Table 1) that amplify a 438 bp DNA fragment of the 16 S rRNA [15] in a 20 µL reaction volume containing 1X DreamTaq buffer, 0.15 mM dNTPs, 1 U DreamTaq polymerase, 0.5 µM each of primers, and 2 µL of template DNA. The volume was made up using nuclease-free water. The cycling conditions were: initial denaturation at 95 ℃ for 5 min, followed by 30 cycles of 95 ℃ for 30 s, 54 ℃ for 30 s, 72 ℃ for 60 s, and a final extension at 72 ℃ for 10 min. *Spiroplasma* was detected by amplifying a 455 bp DNA fragment of the 16 S rRNA gene of the bacterium using 63 F primers (Table 1) as described by Doudoumis et al. [13] in a 20 µL reaction volume consisting of 1X DreamTaq buffer, 0.15 mM dNTPs, 1 U DreamTaq polymerase, 0.25 µM each of primers, and 3 µL of template DNA. The cycling conditions were: initial denaturation at 95 ℃ for 5 min, followed by 30 cycles of 95 ℃ for 30 s, 59 ℃ for 30 s, 72 ℃ for 60 s, and a final extension at 72 ℃ for 10 min. All the PCR products were resolved on 1.5% agarose gel and visualised under UV illumination. The negative control for this study was a double distilled water while *Sodalis*, *Wolbachia* and *Spiroplasma* positive controls were DNA positive samples from a previous study by Mfopit et al. [28].

**Table 1:** Sequence of primers used for molecular identification of organisms

Organism	Primer	Direction	Sequence	Reference
*Glossina* spp	COX1	Forward	TTGATTTTTTGGTCATCCAGAAGT	[23]
*Glossina* spp	COX1	Reverse	TGAAGCTTAAATTCATTGCACTAATC	[23]
*Trypanosoma* spp	ITS-1	External Forward	TGCAATTATTGGTCGCGC	[24]
*Trypanosoma* spp	ITS-1	External Reverse	CTTTGCTGCGTTCTT	[24]
*Trypanosoma* spp	ITS-1	Internal Forward	AAGCCAAGTCATCCATCG	[24]
*Trypanosoma* spp	ITS-1	Internal Reverse	TAGAGGAAGCAAAAG	[24]
*Trypanosoma* spp	gGAPDH	External Forward	TTYGCCGYATYGGYCGCATGG	[25]
*Trypanosoma* spp	gGAPDH	External Reverse	ACMAGRTCCACCACRCGGTG	[25]
*Trypanosoma* spp	gGAPDH	Internal Forward	GCSTAYCAGATGAAGTAC GAC	[26]
*Trypanosoma* spp	gGAPDH	Internal Reverse	GTTYTGCAGSGTCGCCTTGG	[25]
*Sodalis*	pSG2	Forward	TGAAGTTGGGAATGTCG	[27]
*Sodalis*	pSG2	Reverse	AGTTGTAGCACAGCGTGTA	[27]
*Wolbachia*	W-spec	Forward	CATACC TATTCGAAGGGATAG	[15]
*Wolbachia*	W-spec	Reverse	AGCTTCGAGTGAA ACCAATTC	[15]
*Spiroplasma*	63 F	Forward	GCCTAATACATGCAAGTCGAAC	[13]
*Spiroplasma*	63 F	Reverse	TAGCCGTGGCTTTCTGGTAA	[13]

### PCR product purification and sequencing

Amplicons of PCR were excised from the gel and purified using the GeneJET Gel Extraction Kit (Thermo Scientific) following the instructions of the manufacturer. The DNA purified was used for direct Sanger sequencing by a commercial provider (Microsynth SeqLab, Göttingen, Germany). The nucleotide sequences generated were analysed using the Bio-edit software, and a BLAST (Basic Local Alignment Search Tool) search was conducted using Nucleotide BLAST at the NCBI (National Centre for Biotechnology Information) database [29]. Nucleotide sequences isolated in this study were deposited in the NCBI database (GenBank).

### Statistical analyses

Entomological data were expressed in terms of abundance of tsetse flies, estimated by apparent density of flies per trap per day (FTD) according to the following formula: FTD = Nc/TD (where Nc is the total number of captured tsetse flies, T is the total number of traps, and D is the total number of trapping days). Trypanosomes and symbionts hosted by tsetse flies were expressed in terms of prevalence. The Fisher test and the logistic regression model were used to analyse the association between the endosymbionts (*Wolbachia* and *Spiroplasma*) and trypanosome infection in tsetse flies.

## Results and discussion

### Entomological survey

A total of 2,742 tsetse flies were trapped from 5 locations, using 11 biconical traps in three days. These locations include; Magama, Salt-lake, Marshal cave, Kwan Kirya and Bokono (Table 2).

**Table 2:** Relative abundance of Tsetse flies per collection site

Location	Coordinate	Sex of tsetse fly		
		Male	Female	Total
Magama	09°44.870’N/010°30.896’E	375	918	1,293
Salt Lake	09°45.174’N/010°31.508’E	153	370	523
Marshall Cave	09°46.631’N/010°32.296’E	35	79	114
Kwan Kirya	09°45.401’N/010°31.760’E	107	256	363
Bokono Site	09°46.602’N/010°32.361’E	133	316	449
		803	1,939	2,742

**Fig. 2:**
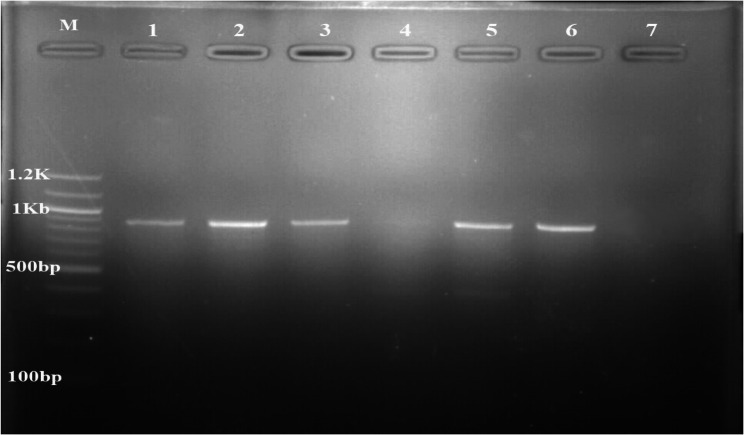
PCR amplification of *Glossina* cytochrome C oxidase 1 gene from gDNA. M: marker (100 bp), lane 1: positive control, 2, 3, 5 and 6: *Glossina tachinoides* samples and lane 4 and 7: negative controls

Out of 2,742 tsetse flies, 803 were males while 1,939 were females. The apparent fly density was 83.09 F/T/D. All the trapped flies were identified morphologically to be *Glossina tachinoides*. A total of 215 were dissected, and found to be teneral. The amplification (Fig. 2) and sequencing of the COX1 partial gene sequence confirmed the *Glossina* species to be *G. tachinoides*. The sequence of our amplicons was closely related (99.87% similarity) to other *Glossina tachinoides* COX1 sequences isolated in the same locality (MG234544, MG234547).

A relatively high apparent tsetse fly density of 83.09 F/T/D was determined. Previous studies reported apparent fly densities of 22.5 [30] and 128.03 F/T/D for *G. tachinoides* [31]. This variation in apparent fly density could be a result of variations in study periods. In our study, samples were collected at the start of the dry season (November), whereas in the other studies, sample collection was in March and August, respectively. We identified a single tsetse fly species (*Glossina tachinoides*), whereas a previous study reported two species: *Glossina morsitans* and *Glossina tachinoides* [30]. The same study reported *Glossina tachinoides* as the predominant species of tsetse flies in Yankari Game Reserve [30]. This difference in *Glossina* species may be due to variation in season and trap locations during the entomological survey.

### Trypanosome infection rate

**Fig. 3:**
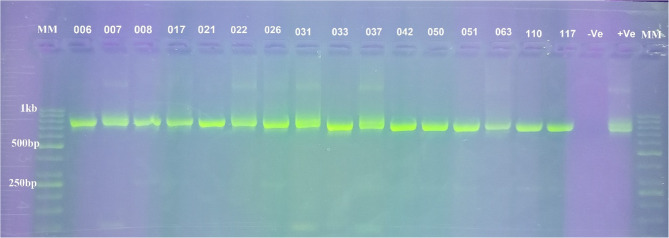
PCR amplification of trypanosome (gGAPDH gene) from genomic DNA of tsetse. MM: marker (50 bp), Lane 6, 8, 17, 21, 22, 26, 33, 42, 50, 51, 63, 117: *T. grayi*, Lane 7, 31 and 37: *T. congolense.* Lane 110: *T. vivax*, -Ve: negative control and + Ve: positive control

Out of 215 guts of *Glossina tachinoides* analysed by ITS-1 nested PCR (Fig. S1), only 23 (10.70%) were infected with at least one species of trypanosomes. Three species of trypanosomes were identified: *Trypanosoma grayi*, *T*. *congolense*, and *T. vivax*. The total number of flies infected by *Trypanosoma grayi*, *Trypanosoma congolense*, *Trypanosoma vivax*,* Trypanosoma grayi/Trypanosoma congolense* mixed infection and *Trypanosoma grayi/Trypanosoma vivax* mixed infection was: 12/215, 1/215, 1/215, 7/215 and 2/215 respectively (Table 3). Following the identification of trypanosomes using ITS-1 nested PCR, gGAPDH nested PCR (Fig. 3) was performed to confirm the trypanosome positive tsetse flies.

**Table 3:** Trypanosome species isolated from midgut of *Glossina Tachinoides*

Type of Infection	T. *grayi*	T. *congolense*	T. *vivax*	T. *grayi/congolense*	T. *grayi/vivax*
Infections (Out of 215)	12/215	1/215	1/215	7/215	2/215
Prevalence	5.58%	0.47%	0.47%	3.26%	0.93%

Overall prevalence of trypanosome infection = 23/215 (10.70%)

The trypanosome infection rate of 10.70% was similar to the 11.9% infection rate obtained in another study in the same location on *Glossina tachinoides* [31]. This study reported the presence of three species of trypanosomes: *T. congolense*, *T. vivax*, and *T. grayi*, which supports the work of Weber et al. [26], who reported the same species. No trypanosome species of the *T. brucei* complex was found, which is in agreement with Weber et al. [26] that reported the absence of *T. brucei* species. This could be as a result of low circulation of species of the *Trypanosoma brucei* complex. *Trypanosoma vivax* was found in only two tsetse fly samples. This low infection rate observed could be attributed to the fact that DNA was isolated only from the midgut of tsetse flies since the development of *T*. *vivax* is restricted to the mouthparts, though few reports indicate that this parasite can be detected from the midgut of tsetse flies up to four days after a blood meal infected with trypanosomes [32].

### Symbiont occurrence rate

**Fig. 4:**
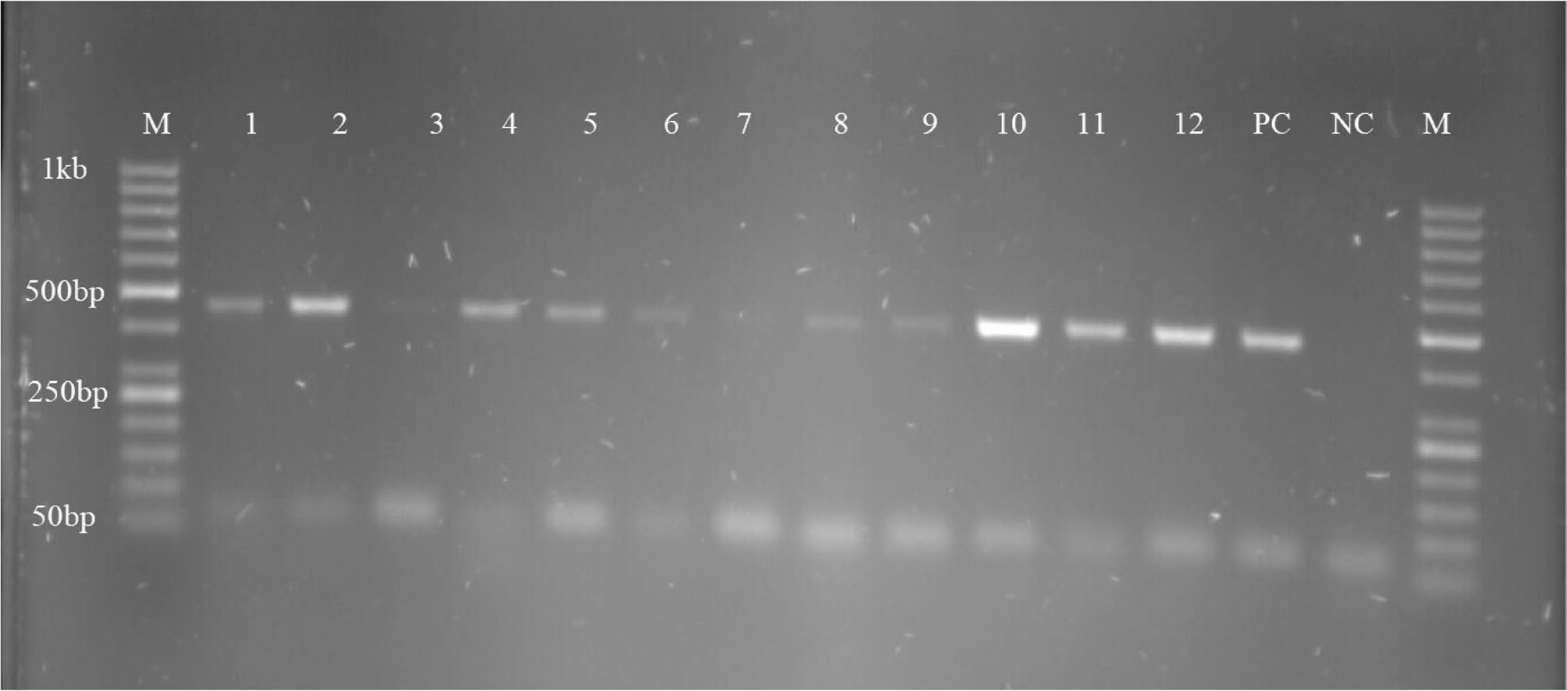
PCR amplification of *Spiroplasma* 16 S rRNA gene M: marker, lane 1 to 12: positive *Spiroplasma* samples, lane 3: *Spiroplasma* negative sample PC: positive control and NC: negative control

Out of 215 midgut samples screened for *Spiroplasma*, *Wolbachia*, and *S. glossinidius* (Fig. 4, Fig. S2), 87 harboured *Spiroplasma*, and 6 harboured *Wolbachia* while no sample was found to harbour *S. glossinidius*, giving endosymbiont infection rates of 40.47%, 2.80% and 0.00% respectively.

Our findings reported the presence of two Tsetse’s symbiotic bacteria: *Spiroplasma* and *Wolbachia*. The absence of *Sodalis glossinidius* in *Glossina tachinoides* agrees with the findings of Mfopit et al. [28], who reported a 0.00% prevalence of *Sodalis* in the same study location, but differs with 3.7% and 16% infection rates reported for *G. austeni* and *G. pallidipes*, respectively, in Shimba Hills National Reserve, Kenya [33]. Findings from our study also differ with the 37.0% *Sodalis* infection rate found in *Glossina tachinoides* captured in Cameroon [6]. The remarkable difference observed with our study could be attributed to differences in tsetse species and geographic location because the microbiota of tsetse flies vary with *Glossina* species and geographic location [14]. The low occurrence of *Wolbachia* (2.80%) corroborates with another study that reported the absence of *Wolbachia* in the *Glossina palpalis* group [34]. However, Kame-Ngasse et al. [6] reported a high prevalence of 68.1% in *Glossina tachinoides* from the Adamawa region, Cameroon. These observations suggest that the prevalence of *Wolbachia* may depend on the ecological conditions of the tsetse fly populations [35]. The high rate of *Spiroplasma* infection in *Glossina tachinoides* (40.47%) is consistent with other studies that reported 37.5% [13] and 44.5% infection rate of *Spiroplasma* in *G. tachinoides* captured from Burkina Faso [34].

### Relationship between trypanosome infection and symbiont presence

Of the 87 tsetse flies harbouring *Spiroplasma*, 13 had at least one species of trypanosome (Table 4). Of the 128 tsetse flies not harbouring *Spiroplasma*, 10 were harbouring trypanosomes. There was no association (*p* = 0.116) between the presence of *Spiroplasma* and the trypanosome infection. Out of 6 flies harbouring *Wolbachia*, 5 had at least one species of trypanosome, while one was trypanosome negative. Of the 209 *Wolbachia* negative flies, 18 had trypanosome infection. The Fisher test (Table 4) shows a positive association between *Wolbachia* presence and trypanosome infection (*p* = 0.001).

**Table 4:** Statistical association of *Wolbachia and Spiroplasma* endosymbionts with trypanosome DNA in *Glossina Tachinoides*

Spiroplasma and trypanosome co-infection (*N* = 215)			Wolbachia and trypanosome co-infection (*N* = 215)		
T/S	S-	S+	W/T	W-	W+
T-	118	74	T-	191	1
T+	10	13	T+	18	5
*p* = 0.116			*p* = 0.001		

T+/T-: Trypanosome positive/negative, W+/W-: *Wolbachia* positive/negative, Sp+/Sp-: *Spiroplasma* positive/negative

An association was not seen between *Spiroplasma* and trypanosome infection, indicating that *Spiroplasma* does not affect the establishment of trypanosomes in the midgut of *Glossina tachinoides*. An association was observed between *Wolbachia* and trypanosomes in tsetse flies, but this relationship could not be conclusive because only a few tsetse flies (6/215) were harbouring the bacterium. This study disagrees with the study of Kante et al. [36], who reported the absence of an association between *Wolbachia* and trypanosomes in *Glossina palpalis palpalis* populations from three sleeping sickness foci of southern Cameroon. The low prevalence of *Wolbachia* could be because of the circulation of another *Wolbachia* strain which could not be detected by the primers used for this study. The study of [37] used the Wsp primers targeting *Wolbachia* surface protein for the screening of *Wolbachia* while we used the W-spec primers targeting the *Wolbachia* 16 S rRNA gene. The study should be performed with different haplotypes of *Wolbachia* to have a clearer picture of the possible relationship existing between *Wolbachia* and the level of infection with trypanosomes in tsetse flies.

## Conclusion

Our study found that *Glossina tachinoides* from Yankari Game Reserve are infected with trypanosomes, with a prevalence of 10.70%. The infection rates of *Sodalis*, *Wolbachia* and *Spiroplasma* were 0.00%, 2.80%, and 40.47%, respectively. An association was observed between *Wolbachia* and trypanosomes in tsetse flies, but no association was observed between *Spiroplasma* and trypanosomes. Findings from this study provide useful data on the microbiota of tsetse flies and could be further used to investigate and understand the role of these symbiotic bacteria on the physiology of tsetse flies, thus helping in the development of new disease control techniques.

## Supplementary Information

Supplementary Material 1: Fig. S1. PCR amplification of trypanosome (ITS-1 gene) from genomic DNA of tsetse fly. MM: marker (50bp), lane 005: mixed infection of T. vivax and T. grayi, lane 007: T. congolense, lane 017: T. grayi, lane 22: mixed infection of T. grayi and T. congolense, lane 037: mixed infection of T. grayi and T. vivax NC: negative control and PC: positive control.

Supplementary Material 2: Fig. S2. PCR amplification of endosymbionts. A. Wolbachia 16S rRNA gene. M: marker, lane 1, 2, 3 and 4 are positive Wolbachia samples, NC: negative control and PC: positive control.

## Data Availability

The datasets supporting the conclusions of this article are included within the article and its additional files. Nucleotide sequences are openly available in National Center for Biotechnology Information (https://www.ncbi.nlm.nih.gov/), with following reference numbers: Glossina tachinoides: OQ653471; Trypanosoma congolense: OQ658682 and OQ658683; Trypanosoma vivax: OQ658688; Trypanosoma grayi: OQ658685, OQ658686, and OQ658687; Wolbachia: OQ658372 and Spiroplasma: OQ658371.
