# Supplementary figures and images for: Spiroplasma, Wolbachia, Sodalis and trypanosome associations in Glossina Tachinoides from Yankari game reserve, Nigeria
Source: BMC Vet Res. 2025 Aug 13;21:514. doi: 10.1186/s12917-025-04959-7 (PMC12344826; doi:10.1186/s12917-025-04959-7)

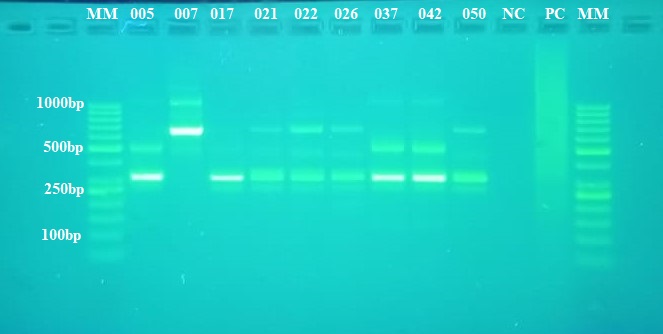

Supplement: Supplementary file 1 — Supplementary Material 1: Fig. S1. PCR amplification of trypanosome (ITS-1 gene) from genomic DNA of tsetse fly. MM: marker (50bp), lane 005: mixed infection of T. vivax and T. grayi, lane 007: T. congolense, lane 017: T. grayi, lane 22: mixed infection of T. grayi and T. congolense, lane 037: mixed infection of T. grayi and T. vivax NC: negative control and PC: positive control. [file 12917_2025_4959_MOESM1_ESM.jpg]

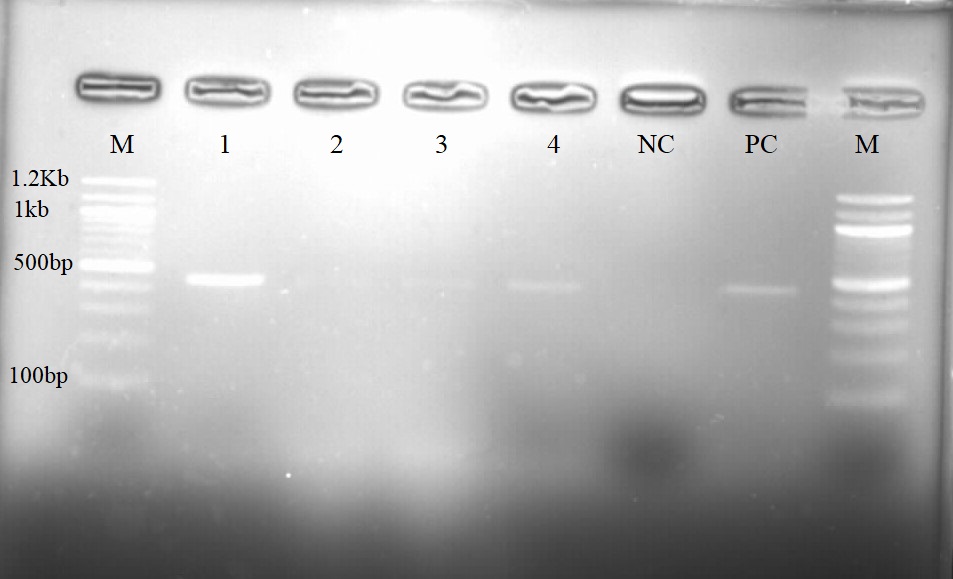

Supplement: Supplementary file 2 — Supplementary Material 2: Fig. S2. PCR amplification of endosymbionts. A. Wolbachia 16S rRNA gene. M: marker, lane 1, 2, 3 and 4 are positive Wolbachia samples, NC: negative control and PC: positive control. [file 12917_2025_4959_MOESM2_ESM.jpg]
